# Application of untargeted liquid chromatography-mass spectrometry to routine analysis of food using three-dimensional bucketing and machine learning

**DOI:** 10.1038/s41598-024-67459-y

**Published:** 2024-07-18

**Authors:** Jule Hansen, Christof Kunert, Hella Münstermann, Kurt-Peter Raezke, Stephan Seifert

**Affiliations:** 1 Institute of Food Chemistry, Hamburg School of Food Science, University of Hamburg, Grindelallee 117, 20146 Hamburg, Germany; 2 Eurofins Food Integrity Control Services GmbH, Berliner Str. 2, 27721 Ritterhude, Germany

**Keywords:** Mass spectrometry, Food profiling, Honey, Routine analysis, Machine learning, Analytical chemistry, Mass spectrometry

## Abstract

For the detection of food adulteration, sensitive and reproducible analytical methods are required. Liquid chromatography coupled to high-resolution mass spectrometry (LC-HRMS) is a highly sensitive method that can be used to obtain analytical fingerprints consisting of a variety of different components. Since the comparability of measurements carried out with different devices and at different times is not given, specific adulterants are usually detected in targeted analyses instead of analyzing the entire fingerprint. However, this comprehensive analysis is desirable in order to stay ahead in the race against food fraudsters, who are constantly adapting their adulterations to the latest state of the art in analytics. We have developed and optimized an approach that enables the separate processing of untargeted LC‑HRMS data obtained from different devices and at different times. We demonstrate this by the successful determination of the geographical origin of honey samples using a random forest model. We then show that this approach can be applied to develop a continuously learning classification model and our final model, based on data from 835 samples, achieves a classification accuracy of 94% for 126 test samples from 6 different countries.

## Introduction

As global trade expands, so does the scope for food fraud. For the protection of public health and consumer interests, as well as for the proper functioning of the European single market, the European Union (EU) ensures the establishment and maintenance of control standards by accredited laboratories (The Treaty on the Functioning of the EU). The methods used for official controls should be the latest technology and therefore subject to continuous improvement (Regulation (EU) 2017/625).

The current approach for honey, as for other foods, requires a combination of methods for authentication^1^. ^13^C/^12^C stable carbon isotope ratio mass spectrometer coupled with elemental analyzer- and liquid chromatography (δ13C‑EA/LC‑IRMS)^2^, untargeted proton nuclear magnetic resonance (^1^H­NMR)^3^, targeted LC‑HRMS^4^, pollen microscopy (pollen identification and determination of the relative pollen content, DIN 10,760 mod. (2002–05)) and sensory (ICS SOP 520–02 (2018–08)) are currently being used to detect syrup adulterations, determine the geographical and botanical origin of honey, and monitor quality parameters. Although this combination of methods provides a high level of confidence for authentication, it is quite comprehensive and complex. A major challenge for the classification of the geographical origin is that the samples within a country can be very diverse, for example due to different varieties, several regions within countries and changing weather conditions between harvest years, making the long-term application of targeted methods very difficult. With regard to syrup addition, the variant marker databases for targeted analysis must constantly be updated, as fraudsters are aware of the substances being tested and develop specific fraud methods^5^. Furthermore, tailored sugar syrups that replicate the chemical composition of the primary sugars in honey are used, which is why falsifications are not necessarily detected by targeted analysis^3^. Commercial laboratories therefore need a sensitive untargeted screening method to keep up and detect deviating samples even before the specific adulteration is defined. ^1^H-NMR and LC‑HRMS have the potential to be such an untargeted screening method, applicable for the classification of various food^6–12^. The untargeted application of ^1^H­-NMR is already possible in routine analysis because the method is very robust and reproducible meaning that data obtained from different devices over a long period of time are comparable^13^. In addition, ^1^H­NMR data are processed in such a way that the features in different spectra, which were not necessarily processed simultaneously, can be matched. This is highly valuable as it is an important basic requirement to create large datasets for authentication. However, due to the low sensitivity, e.g. compared to mass spectrometric approaches, ^1^H­NMR cannot be used as a single authentication method.

Furthermore, this is demonstrated by the detection of syrup in honey, as this is only possible in relatively high proportions^14^. LC-HRMS, on the other hand, is characterized by a very high sensitivity but the other criterion for routine applications, robustness, is unfortunately not met without careful quality control procedures^15–18^. The lack of robustness is mainly caused by the varying age and degree of contamination of the devices and, in particular, the columns used^18^. This leads to a slightly different interaction between the compounds and the columns and, hence, result in measurement-based differences in the spectra if they are not obtained at the same time with the same device^19^. Because of these variations, current LC‑MS data processing workflows like vendor specific software (e.g. Compound Discoverer™ Software^20^) or the framework for processing and visualizing LC‑MS data xcms^21–23^ include a so-called retention time alignment step. Additionally, in common data processing strategies, a correspondence step is applied to match the detected and aligned chromatographic peaks between the different spectra to generate a common peak list, which is unique for each analysis. Hence, it is currently not possible to analyze untargeted LCMS spectra that were processed separately. For long‑term application, this means an immense computational effort, as the entire data set would have to be reprocessed each time new authentic data is added or a newly analyzed sample is to be classified. It is therefore currently very difficult to use untargeted LC-HRMS in a commercial laboratory, where several hundred samples are analyzed every week.

When such untargeted screening methods are applied for food authentication, high-dimensional data are produced that contain many variables, which must be evaluated in an appropriate manner. For this, usually machine learning methods are applied on training data to obtain a model based on specific differences between predefined classes, e.g. regarding the geographical origin. This model must be validated with data from samples that are not part of the training data and can subsequently be applied for the classification of other samples, e.g. with unknown or questionable origin. There are several algorithms that can be applied to train classification models for untargeted screening methods, e.g. support vector machines (SVM), artificial neural networks (ANN) and random forest (RF).

RF is a non-parametric ensemble learning algorithm, which is based on multiple binary decision trees. Because each of these trees is trained on a subset of the samples, called bootstrap sample, the remaining, out-of-bag samples are used to generate an independent (out-of-bag, OOB) error that is equivalent to the use of independent validation data^24,25^. This and other advantages, such as easy adaptation to imbalanced training data, make RF a powerful algorithm for the classification of LC-HRMS data of food, one of the reasons why it is widely used for this purpose^6,8,11,26,27^.

Here we present our new data processing approach called BOULS (bucketing of untargeted LCMS spectra), which is specifically adapted to the routine analysis of food. It is based on the established xcms workflow in R but allows the analysis of data obtained from different devices and at different points in time that was not necessarily processed together. This is achieved through the constant use of a central spectrum for retention time alignment and a bucketing step in which the spectrum is divided into buckets summing up the total intensity of the signals. In contrast to bucketing of NMR data and a previous approach for LC–MS data processing^28^, the LCMS buckets obtained by our approach have three dimensions (retention time, mass-to-charge-ratio (m/z) and feature intensity). As a result, newly acquired spectra can be classified and added to the random forest training dataset in a simple and fast way, as no re-evaluation of the entire data and no batch correction^29,30^, feature identification^31^ or feature matching^32^ is required for successful classificaton. For implementation, optimization and validation, the approach was applied for the determination of the geographical origin of honey over longer periods of time and individual parameters of the workflow were optimized using Design of Experiments (DOE). Finally, we show the long-term application in a routine laboratory to build increasingly accurate random forest classification models, which are also able to react dynamically to changes in the respective groups.

## Methods

### LC-HRMS analysis

The honey samples were analyzed using three LC-HRMS devices comprising Thermo Scientific™ UltiMate™ 3000 systems coupled to Thermo Scientific™ Q Exactive™ Hybrid Quadrupole-Orbitrap™ High-Resolution Mass Spectrometers. Each sample was measured with two different Liquid Chromatography (LC) approaches. Due to preliminary tests, hydrophilic interaction liquid chromatography (HILIC, Accucore-150-Amide-HILIC 150 × 2,1) in negative ion mode was applied to analyze polar compounds while non-polar compounds were analyzed by reverse phase (RP, Hypersil Gold C18* 150 × 2,1) chromatography in positive ion mode. This setup generated two different types of data for each sample, which will be referred to as HILIC and RP data. For both, the mobile phases water and acetonitrile with acetic acid as modifier were used. For the normalization strategy ISTD_add (see following subsection), a third mobile phase with 2% sorbic acid in positive ion mode (RP method) and 10% in negative ion mode (HILIC method) in an acetonitrile–water-mixture (50/50, v/v) with acetic acid as additive was isocratically injected. The different sorbic acid concentrations were chosen due to missing signals in the spectra obtained in negative ion mode.

The ionization of the chromatographically separated compounds was performed using electrospray ionization (ESI) and the data was acquired in profile mode. The mass spectrometric analysis was conducted using the variable data-independent acquisition (vDIA) method developed by Thermo Scientific™ Orbitrap™. vDIA is a data acquisition method that utilizes MS/MS precursor isolation windows of differing mass ranges, covering the entire mass range of the preceding full scan^33^. For the data analyzed in this study, the full scan comprised 100–1500 Da (MS1). This range was covered by six isolation windows, in which ions of the masses 150, 250, 350, 450, 750 and 1250 Da were fragmented respectively (MS2). The respective fragments were detected in the ranges 50–225, 50–330, 50–430, 50–535, 69–1045 and 104–1555 Da^4^.

### Data processing with BOULS

**Figure 1:**
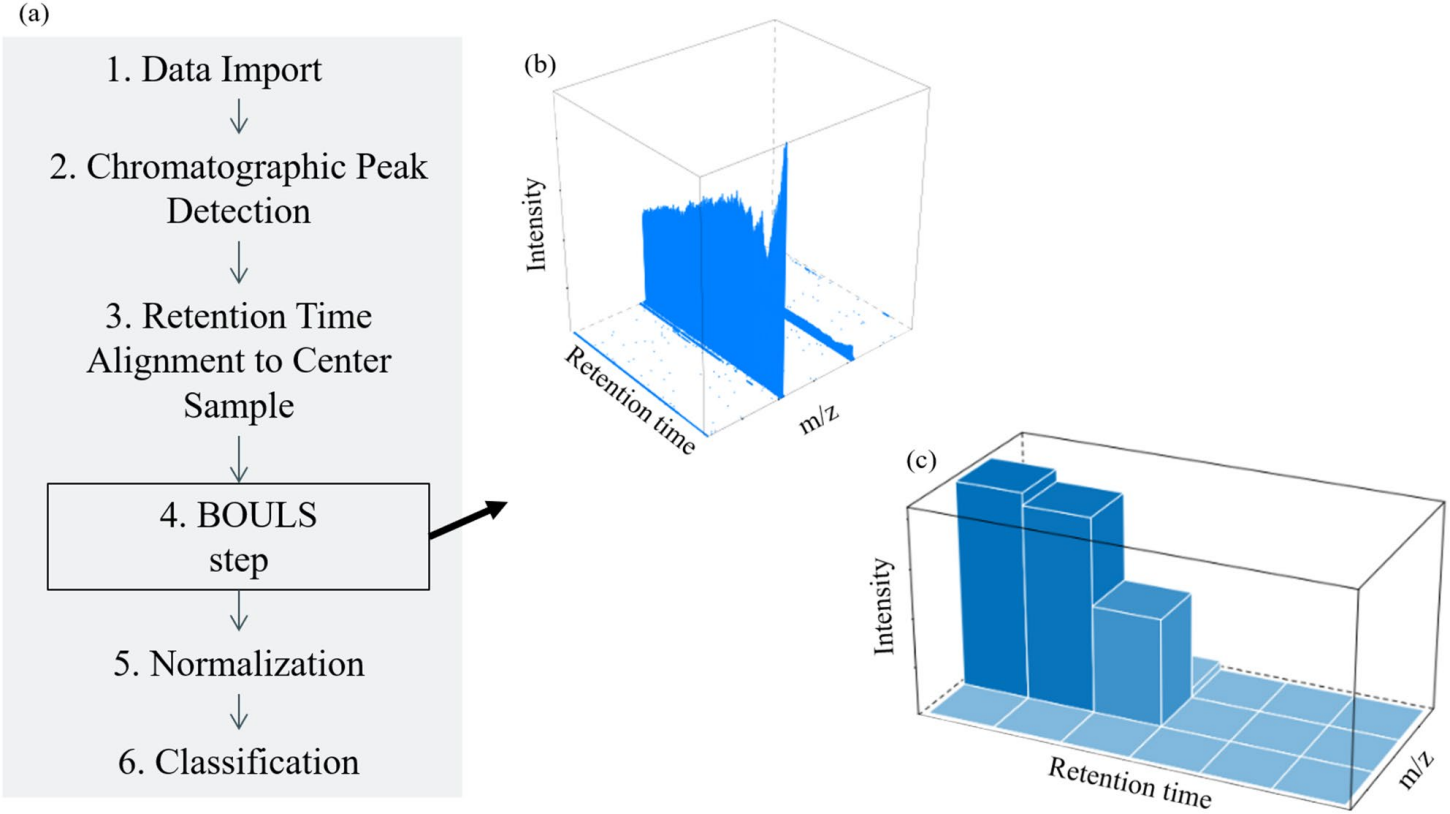
(**a**) Overview of the novel data processing approach for the analysis of untargeted LC-HRMS data in routine analysis: A new step called BOULS replaces the correspondence step in previous workflows. To introduce this step, an example LC-HRMS spectrum after retention time alignment is shown in (**b**). Subsequently, the same spectrum after the application of BOULS summing up the signals in the respective buckets is depicted in (**c**).

The developed BOULS approach is schematically illustrated in Fig. 1 and published at https://github.com/AGSeifert/BOULS (requires Linux OS). It is based on the established xcms workflow in R and uses the same functions for data import and chromatographic peak detection^34^.

For data import (step 1 in Fig. 1), the thermo-specific raw files are first converted to open-format mzML files using MSConvert, which is part of the ProteoWizard software package (Version: 3.0.21078-7da1f1136 (developer build)). Here, the filter peakPicking is used to convert the profile mode data into centroided data^35^. Subsequently the data is imported into R using the Bioconductor package mzR^35–39^ (version 2.26.0) storing information about the samples and analyses, such as name, geographical origin, instrument and method. The package MSnbase^40,41^ (version 2.18.0) is then used to load and store the data in an object that is compatible with the xcms package^21–23^ (version 3.14.0). The chromatographic peak detection (step 2 in Fig. 1) is performed by the centWave algorithm^23^ using the parameters peakwidth of 15 s and ppm of 5 Da, which is individual for each experimental set up and which was set according to the extracted ion chromatogram of the internal standards. The retention time alignment (step 3 in Fig. 1) is performed by using the obiwarp method^42^, whereby the parameters binSize and localAlignment are set to 0.1 and TRUE, respectively. The spectrum with the most peaks was used as center spectrum.

Subsequently, the novel BOULS step (step 4 in Fig. 1) is conducted and the data is divided into 3 dimensional buckets summing up the total intensity of the signals (see Fig. 1b and c). The parameters of this step, namely the bucket size in the retention time (RT) and m/z dimensions, are optimized in a DOE, which is described and evaluated below.

Various approaches have been applied for normalization (step 5 in Fig. 1). For the first normalization strategy, the internal standard in the third mobile phase (ISTD_add) sorbic acid was used and the total intensity of an RT-bucket was divided by the total intensity of all sorbic acid signal intensities. For total ion current (TIC) and base peak chromatogram (BPC) normalization, the summarized intensities of each bucket were divided by the sum of intensities of all signals and the most intensive signal, respectively. For TIC_RT and BPC_RT, the individual RT-buckets were considered separately and the intensity of a bucket was divided by either the sum (TIC_RT) or the maximum (BPC_RT) of the intensities of the respective RT‑bucket. Also, the different normalization approaches were evaluated in a DOE.

For classification (step 6 in Fig. 1), RF was applied using the R package ranger^43^ (version 0.14.1, CRAN) with the parameters ntree = 5000 and the respective default parameters for mtry and min.node.size (square root of the total number of variables and 1, respectively). To compensate for class imbalance, the parameter case.weights was chosen according to the size of the respective classes^43^.

### Data

For the development, validation and application of the BOULS approach, four data sets compiled from samples of customers of a routine laboratory were used. As the successful analysis and comparison over a long period of time are crucial, different time periods were chosen for obtaining the training data and between obtaining the training and test data. For the analysis of each sample set, both, HILIC and RP, were used and the samples were measured in approximately equal proportions using one of the three instruments.

#### Data set 1

To optimize the BOULS approach parameters by a DOE (see section DOE1: Initial optimization), a data set obtained from 123 honey samples (246 spectra) from Argentina (18), Brazil (19), Canada (18), China (8), Ukraine (40) and the USA (20), was analyzed. The measurements were performed over a period of six weeks and the performance was evaluated by the OOB error of the obtained random forests.

#### Data set 2

To test the robustness of the developed approach over time, training and test data measured with a time offset of seven months were analyzed. For training, data set 1 was used. However, since they were not included in the test data, the data from the Chinese samples were omitted. The test set consisted of 27 spectra from 5, 5, 4, 6 and 7 samples from Argentina, Brazil, Canada, Ukraine and the USA, respectively.

#### Data set 3

The third data set was used for the initial implementation of the developed approach in routine analysis. For training, data of 565 samples from Argentina (93), Brazil (65), Canada (9), India (69), Ukraine (250) and the USA (79) were used. The test set consisted of 126 spectra from 8, 11, 65, 1, 13 and 28 samples from Argentina, Brazil, India, Canada, Ukraine, and the USA, respectively.

#### Data set 4

The fourth data set was used to analyze, whether the prediction accuracy improves by extending the model in long-term application. The extended training data set contained data from 835 samples from Argentina (170), Brazil (133), Canada (16), India (126), Ukraine (264), and the USA (126). The resulting model was used to classify the same test data as in the previous section to compare the performance of the two models directly.

### Design of experiment (DOE) for the optimization of the BOULS parameters

In order to optimize the parameters of the BOULS approach, two full factorial DOEs described in the following two subsections were conducted. In each case, the expand.grid function of the R base package^44^ (version 4.2.2) was used to generate a data frame with all possible combinations of the factor levels. After the execution of the workflow, Analysis of Variance (ANOVA) (aov function of the base stats package) in combination with Tukey’s honest significant difference (TukeyHSD function in base R package) was applied to compare the different factors and calculate p-values.

#### DOE1: Initial optimization

Table 1 shows the applied factors together with their respective levels of the first DOE. Bucket sizes for the RT and m/z dimension, as well as the different normalization approaches introduced in the previous section were evaluated.

**Table 1:** Factors and levels of DOE 1 for the initial optimization of applied parameters of the BOULS approach.

Factor	Level	Description
Bucket size RT [s]	5	Size of the LCMS spectrum buckets in RT dimension
	20	
	80	
Bucket size m/z [Da]	2	Size of the LCMS spectrum buckets in mass dimension
	5	
	10	
	20	
Normalization	BPC	Divide by maximum intensity
	BPC_RT	Divide by maximum intensity per RT bucket
	TIC	Divide by sum of intensities
	TIC_RT	Divide by sum of intensities per RT bucket
	ISTD_add	Divide by signal-intensity of the internal standard added to the third mobile phase

#### DOE2: Validation and optimization for long-term application

In order to establish the BOULS approach for long-time application, a second DOE was applied with the factors and levels shown in Table 2. In addition to the already applied factors in DOE 1, which were validated for long-term application, also the addition of fragment (MS2) data and the combination of HILIC and RP data was evaluated. For the former, the fragment RP data was processed with the BOULS step with either small buckets (5 Da and 5 s) or large buckets (20 Da and 80 s) and combined with the full RP data or not. Since the individual isolation windows vary in size (see section LC-HRMS analysis), the exact size of the buckets was adjusted to achieve divisibility. For the latter, the HILIC data was either included with or without the fragment data that was processed as just described or not.

**Table 2:** Factors and levels of the second DOE for validation and optimization for long-term application of the BOULS approach.

Factor	Level	Description
Bucket size RT [s]	5	Size of the LCMS spectrum buckets in RT dimension for RP full scan data
	20	
	80	
Bucket size m/z [Da]	2	Size of the LCMS spectrum buckets in mass dimension for RP full scan data
	5	
Fragments	Yes_small	RP full scan plus RP fragment spectra with a bucket size of 5 s and 5 Da
	Yes_large	RP full scan plus RP fragment spectra with a bucket size of 80 s and 20 Da
	No	Use only RP full scan
Data Fusion	Yes_full	Add HILIC full scan data (bucket sizes according to RP full scan data)
	Yes_full_frag_small	Add HILIC full scan plus HILIC fragment spectra with a segment size of 5 s and 5 Da
	Yes_full_frag_large	Add HILIC full scan plus HILIC fragment spectra with a bucket size of 80 s and 20 Da
	No	No HILIC data included
Normalization	BPC	Divide by maximum intensity
	BPC_RT	Divide by maximum intensity of the respective RT bucket
	TIC	Divide by sum of intensities in the spectrum
	TIC_RT	Divide by sum of intensities in the respective RT bucket

## Results and discussion

This work presents BOULS, a data processing workflow that allows the application of untargeted LC-HRMS for routine analysis. In the following sections, this approach will be applied for the analysis of data from different devices and the BOULS parameters will be analyzed and optimized (first subsection). Subsequently, the long-term applicability with optimized parameters will be demonstrated (second subsection), and an approach for the implementation in routine analysis is shown (third subsection).

### Analysis of data from different devices

For the analysis and optimization of the BOULS parameters, a full factorial DOE for both HILIC and RP data was applied and the results are shown in Table 3. The size of the buckets was optimized for the RT and mass dimension. The analysis of the RT dimension showed that the largest possible bucket size should be used for the HILIC data as significantly lower OOB errors were achieved using the size 80 s compared to 20 s (p < 0.01), 80 s compared to 5 s (p < 0.001) and 20 s compared to 5 s (p < 0.01). On the contrary, the results for the RP data showed that the lowest possible bucket size should be used because significantly lower OOB errors were achieved using the size 20 s compared to 80 s (p < 0.01) and 5 s compared to 80 s (p < 0.001). In principle, the determination of the RT bucket size involves a compromise between compensation for technical differences between the measurements, mainly caused by the use of different devices, and a greater resolution of the information contained in the spectra (small bucket size). The former seems to be more important for the HILIC data, while the latter is more important for the RP data. This confirms the property that HILIC chromatography is less robust than RP chromatography^45^. For the optimization of the bucket size in mass dimension, small values were advantageous for the HILIC data since significantly smaller OOB errors were achieved using the level 2 Da compared to 10 Da (p < 0.05), 2 Da compared to 20 Da (p < 0.001), 5 Da compared to 20 Da (p < 0.001) and 10 Da compared to 20 Da (p < 0.01). For the RP data, also smaller values for this bucket size showed smaller OOB errors but the differences were not significant. The general preference of smaller values for this parameter can be explained by the fact that a greater resolution of the signals results in models based on more detailed information, which improves the classification. Deviations in the mass signals are not present in the same extend as in the retention time and must therefore not be compensated for by high values for this bucket size.

**Table 3:** Results of the initial optimization of the BOULS parameters for the HILIC and RP data based on DOE1.

Factor	Levels combination	Difference in OOB error (HILIC data)	Difference in OOB error (RP data)
Bucket size RT [s]	*20*/**5**	− 1.0**	0.18
	*80*/**5**	− 2.0***	1.2***
	*80*/**20**	− 0.98**	1.1**
Bucket size m/z [Da]	5/***2***	0.19	0.13
	10/***2***	1.0*	0.62
	20/***2***	2.3***	0.54
	10/***5***	0.81	0.48
	20/***5***	2.1***	0.41
	**20**/*10*	1.3**	− 0.081
Normalization	***TIC***/BPC	− 0.54	− 0.54
	ISTD_add/***BPC***	0.54	4.2***
	*BPC_RT*/**BPC**	− 0.41	0.51
	*TIC_RT*/**BPC**	− 1.3*	0.17
	ISTD_add/***TIC***	1.1	4.7***
	BPC_RT/***TIC***	0.14	1.1
	*TIC_RT*/**TIC**	− 0.75	0.71
	***BPC_RT***/ISTD_add	− 0.95	− 3.7***
	***TIC_RT***/ISTD_add	− 1.8***	− 4.0***
	***TIC_RT***/BPC_RT	− 0.88	− 0.34

The respective level reaching the lower OOB error is in italics and bold for the analysis of HILIC and RP data, respectively. The third and fourth columns show the differences between the averaged classification accuracies for the HILIC and RP data, respectively.

*p < 0.05.

**p < 0.01.

***p < 0.001.

The comparison of the individual normalization approaches shows that the use of an internal standard in ISTD_add generally results in significantly higher OOB errors for the RP data (p < 0.001 for all comparisons) and higher or significantly higher errors for the HILIC data. Since this normalization is obviously inferior to the others, it was neglected for the workflow and the following analyses. The other normalization approaches mostly do not show significant differences, which is why all of them were further evaluated. Table 4 shows the confusion matrix for the classification of the geographical origin of honey using the best parameter combination for each of the two data sets. Classification based on the RP data results in more accurate results with an overall OOB-error of 5% compared to 10% using the HILIC data. This is particularly evident in the classification of samples from the USA and Brazil and is probably due to the lower robustness of the HILIC method.

**Table 4:** Confusion matrix for the RF model using HILIC (first value) and RP (second value, bold) data achieving an OOB error of 10 and 5%, respectively.

	predicted					
**true**	Argentina	Brazil	Canada	China	Ukraine	USA
Argentina	13/**14**	1/**0**	0/**0**	1/**0**	1/**1**	2/**3**
Brazil	1/**0**	17/**19**	0/**0**	0/**0**	0/**0**	1/**0**
Canada	0/**0**	0/**0**	17/**18**	0/**0**	0/**0**	1/**0**
China	0/**0**	0/**0**	0/**0**	8/**8**	0/**0**	0/**0**
Ukraine	0/**1**	0/**0**	0/**0**	0/**0**	40/**39**	0/**0**
USA	0/**0**	1/**0**	2/**1**	0/**0**	1/**0**	16/**19**

The HILIC data was processed with the bucket sizes 80 s and 5 Da and the normalization strategy TIC, while the RP data was processed with the bucket sizes 5 s and 2 Da and the normalization strategy TIC.

The overall high classification accuracy demonstrates the general applicability of our approach using bucketed spectra of different devices over six weeks, a comparatively short period of time. In the next section, we will further optimize data processing parameters and analysis for the application of the approach over a longer period of time.

### Validation and optimization of the BOULS approach for long-term application

For practical application of machine learning approaches in routine analysis, a model is usually trained with currently available data and applied to data that is obtained at a much later point in time from samples that are then to be classified. Therefore, we evaluated BOULS and its parameters under these conditions in a second DOE. In addition to the parameters of the bucket sizes and normalization strategies, we also analyzed here, whether a fusion of the RP data with the HILIC data and an addition of the MS2 fragment data would be advantageous for long-term application. The results are summarized in Table 5.

**Table 5:** Results of DOE 2 for long-term application of BOULS.

Factor	Levels combination	Difference in classification error
Bucket size RT [s]	**20**/5	− 0.73
	**80**/5	− 1.8
	**80**/20	− 1.1
Bucket size m/z [Da]	5/**2**	1.41*
Normalization	**TIC**/BPC	− 4.83***
	BPC_RT/**BPC**	9.2***
	**TIC_RT**/BPC	− 1.0
	BPC_RT/**TIC**	14.0***
	TIC_RT/**TIC**	3.81***
	**TIC_RT**/BPC_RT	− 10.2***
Fragments	**Yes_large**/yes_small	− 1.5
	**No**/yes_small	− 1.3
	No/**yes_large**	0.15
Data Fusion	**Yes_full_frag_large**/yes_full	− 2.1
	**Yes_full_frag_small**/yes_full	− 1.4
	No/**yes_full**	5.1
	**Yes_full_frag_small**/yes_full_frag_large	− 1.4
	No/**yes_full_frag_large**	5.1
	No/**yes_full_frag_small**	1.5

The level reaching the lower classification error for the test data is shown in bold in the second column and the third column shows the differences between the classification errors.

*p < 0.05.

**p < 0.01.

***p < 0.001.

Analysis of bucket size showed, that there were no significant differences between the bucket sizes in the RT dimension while at the mass dimension the bucket size of 2 Da resulted in significantly lower classification errors (p < 0.05). For the evaluation of the normalization, using the sum of all signal intensities (TIC) significantly outperformed all of the other approaches (p < 0.001). No significant differences were observed for adding RP MS2 data (factor: fragments) or HILIC data with or without fragment data (factor: data fusion), even though there were differences in classification error of up to 5%. Based on these results, there would be two options for selecting the optimum parameters for further application. First, with view to computational effort and the aim of having a model that is as simple as possible, only RP data could be used. However, since we observed differences that could become significant in the long run, especially when data of new classes are added, we decided to evaluate the parameter combinations that provide the best classification results (see Supplementary Table S1). Here it can be seen that both the lowest error for the test data of 15% and for the training data of 7% is achieved with the parameter combination, using bucket sizes of 20 s and 2 Da for the RP MS1 data, a TIC normalization, large buckets of 80 s and 20 Da for the RP MS2 fragment and to include the HILIC MS1 data with equal bucket sizes as for RP MS1 and with HILIC MS2 data with small buckets of 5 s and 5 Da (confusion matrix of the test data in Supplementary Table S2). In general, the high classification accuracy shows that the use of LC-HRMS over a longer period of time is possible in a routine laboratory if the BOULS approach is applied.

### Implementation in routine analysis

**Figure 2:**
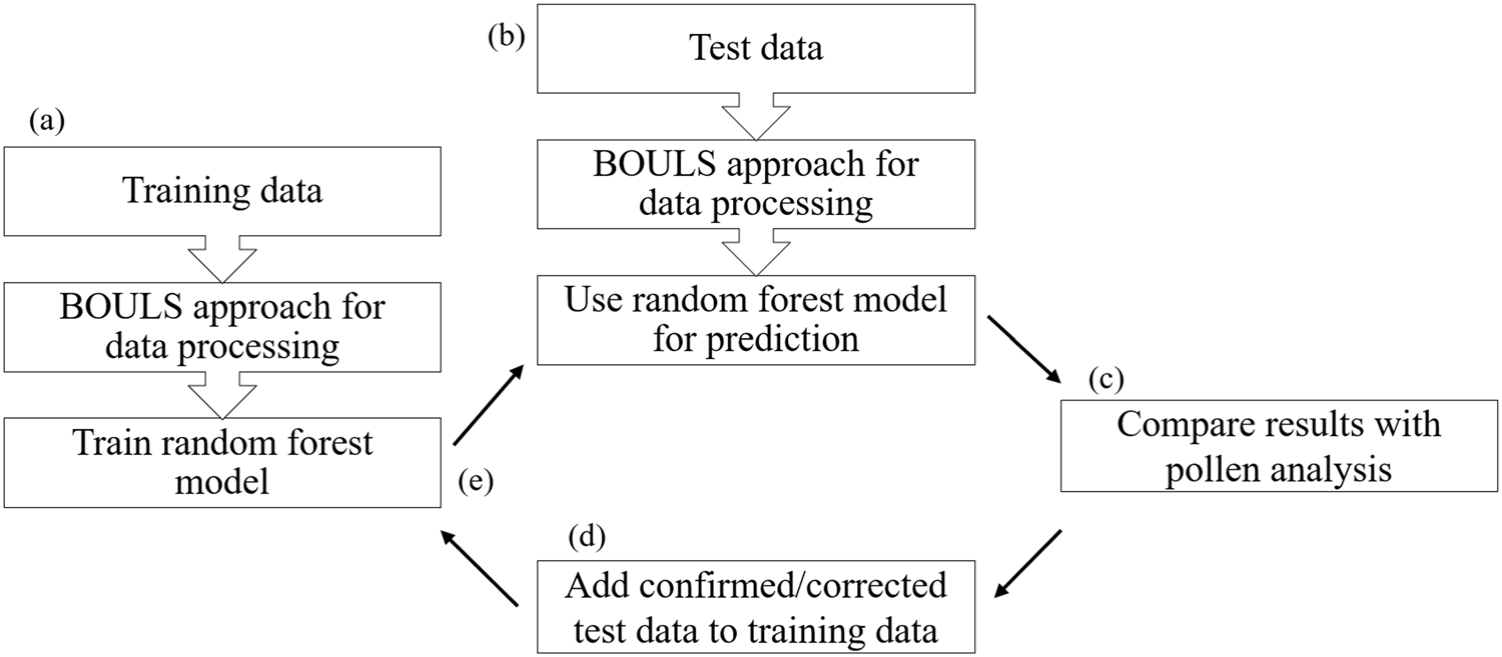
Schematic overview illustration of the application of the BOULS approach in routine analysis.

Having demonstrated the general applicability of BOULS for analyzing LC-HRMS data from multiple devices over a long period of time and defined a parameter combination for the application, we will now use the example of determining the geographical origin of honey to show how it can be used for routine analysis of food in a commercial laboratory. The general procedure is shown in Fig. 2.

An initial dataset is processed with BOULS and used to train an RF classification model. Subsequently, data of new samples with unknown geographic origin are processed separately and, the geographic origin of these test data is predicted by the model. The results of the prediction are compared to the results of reference methods like pollen analysis to evaluate the model. The data from the sample, including the correct class assignment, is then added to the training data, which is very simple because the data is processed into equal buckets with BOULS instead of using the correspondence step applied in other workflows. Finally, a new RF model based on the extended training data is trained and used for the prediction of the subsequent samples. In this way, continuous learning models are developed that increasingly reflect the entire variance of the relevant groups. This is shown in the example in Table 6, where we predicted the same test data with a growing model. The improved model, based on the larger training data set, achieved an 11% higher overall accuracy, mainly due to a better prediction of the American and Brazilian samples.

**Table 6:** Confusion matrix for the prediction of the test data with the initial RF model based on data from 565 samples (first value) and the extended RF model based on data from 835 samples (second value, bold) achieving an overall accuracy of 83% and 94%, respectively.

	predicted					
**true**	Argentina	Brazil	India	Canada	Ukraine	USA
Argentina	8/**8**	0/**0**	0/**0**	0/**0**	0/**0**	0/**0**
Brazil	0/**0**	5/**11**	0/**0**	0/**0**	0/**0**	6/**0**
India	0/**0**	2/**1**	61/**62**	0/**0**	0/**0**	2/**2**
Canada	0/**0**	0/**0**	0/**0**	0/**0**	0/**0**	1/**1**
Ukraine	0/**0**	0/**0**	0/**0**	0/**0**	13/**13**	0/**0**
USA	1/**2**	4/**2**	4/**0**	0/**0**	1/**0**	18/**24**

However, the only Canadian samples is still not classified correctly when the extended model is used. Since also the extended model is only based on 16 Canadian samples, it is reasonable to assume that a correct classification can also be achieved here if the entire variance of this class is represented by a correspondingly higher number of samples.

## Conclusions

In this study, we have presented the BOULS approach for LCMS data processing making it possible to use data from samples measured on different devices and at different times. This is achieved through the generation of a standardized data structure and enables the training of large machine learning models for routine analysis in commercial laboratories. We have demonstrated this application here for the determination of the geographical origin of honey, which is a highly contaminating matrix for LCMS analyses. Since this characteristic strongly influences the comparability of the spectra and the use of BOULS obviously makes this possible anyway, we assume that this approach can be applied to a wide range of different foods and other biological samples. Most of the parameters of the workflow proved to be quite robust in our comparisons, which greatly simplifies this expansion of the application. Furthermore, it is also possible to extend the approach to analyze liquid chromatography-ion mobility spectrometry-mass spectrometry (LC-IMS-MS) data, thereby incorporating even more information into the classification models.

### Supplementary Information

Supplementary Information.

## Data Availability

The datasets analyzed during the current study are not publicly available as they were generated from samples from customers of a commercial laboratory. However, the BOULS approach is published in an R package here: https://github.com/AGSeifert/BOULS and example data is provided here: https://www.fdr.uni-hamburg.de/record/13535.
