# Supplementary material for: Application of untargeted liquid chromatography-mass spectrometry to routine analysis of food using three-dimensional bucketing and machine learning
Source: Sci Rep. 2024 Jul 18;14:16594. doi: 10.1038/s41598-024-67459-y (PMC11258308; doi:10.1038/s41598-024-67459-y)
Supplement: Supplementary file 1 — Supplementary Information. [file 41598_2024_67459_MOESM1_ESM.zip › SupplementaryInformation_BOULS.doc]

**Supplementary information**

**Application of untargeted liquid chromatography-mass spectrometry to routine analysis of food using three-dimensional bucketing and machine learning**

**Authors:** Jule Hansen^1^, Christof Kunert^2^, Hella Münstermann^1^, Kurt-Peter Raezke^2^, Stephan Seifert^1^

**Affiliations:**

^1^Institute of Food Chemistry, University of Hamburg, Hamburg, Germany

^2^Eurofins Food Integrity Control Services GmbH, Ritterhude, Germany

**Supplementary tables**

**Supplementary Table S1:** Results of the parameter optimization in DOE2 showing the top 10 parameter combinations with the smallest classification error for the test data.

**Supplementary Table S2:** Confusion matrix for the classification of the test data achieving a classification error of 15%. The optimized parameters 20 s and 2 Da for the RP MS1 data, a TIC normalization, large buckets of 80 s and 20 Da for the RP MS2 fragment data in combination with HILIC MS1 data with equal bucket sizes as for RP MS1 and HILIC MS2 data with small buckets of 5 s and 5 Da were used.

**Supplementary Table S1**

| **Bucket size RT [s]** | **Bucket size**  **m/z [Da]** | **Normalization** | **Fragments** | **Data Fusion** | **Error test data**  **[%]** | **OOB error**  **RF model [%]** |
| --- | --- | --- | --- | --- | --- | --- |
| 20 | 2 | TIC | Yes_large | Yes_full_frag_small | 15 | 7 |
| 80 | 2 | BPC | no | Yes_full | 19 | 10 |
| 20 | 5 | TIC | Yes_large | Yes_full_frag_large | 19 | 7 |
| 80 | 2 | BPC | no | Yes_full_frag_large | 19 | 10 |
| 5 | 5 | TIC | No | Yes_full_frag_small | 19 | 7 |
| 80 | 2 | BPC | Yes_small | Yes_full | 22 | 10 |
| 80 | 2 | BPC | Yes_large | Yes_full | 22 | 9 |
| 80 | 5 | BPC | Yes_large | Yes_full | 22 | 8 |
| 5 | 2 | TIC | Yes_large | Yes_full | 22 | 7 |
| 80 | 2 | TIC | Yes_large | Yes_full | 22 | 9 |

**Supplementary Table S2**

|  | predicted |  |  |  |  |
| --- | --- | --- | --- | --- | --- |
| true | Argentina | Brazil | Canada | Ukraine | USA |
| Argentina | 5 | 0 | 0 | 0 | 0 |
| Brazil | 0 | 5 | 0 | 0 | 0 |
| Canada | 0 | 0 | 4 | 0 | 0 |
| Ukraine | 0 | 1 | 0 | 5 | 0 |
| USA | 0 | 1 | 2 | 0 | 4 |
